# Multispectral optoacoustic tomography of benign parotid tumors in vivo: a prospective observational pilot study

**DOI:** 10.1038/s41598-024-61303-z

**Published:** 2024-05-08

**Authors:** Mussab Kouka, Maximilian Waldner, Orlando Guntinas-Lichius

**Affiliations:** 1 Department of Otorhinolaryngology, Jena University Hospital, Am Klinikum 1, 07747 Jena, Germany; 2 Department of Medicine, University of Erlangen-Nuremberg, 91054 Erlangen, Germany

**Keywords:** Multispectral optoacoustic tomography (MSOT), Pleomorphic adenoma, Warthin tumor, Hemoglobin, Head and neck cancer, Photoacoustics

## Abstract

Parotid lumps are a heterogeneous group of mainly benign but also malignant tumors. Preoperative imaging does not allow a differentiation between tumor types. Multispectral optoacoustic tomography (MSOT) may improve the preoperative diagnostics. In this first prospective pilot trial the ability of MSOT to discriminate between the two most frequent benign parotid tumors, pleomorphic adenoma (PA) and Warthin tumor (WT) as well as to normal parotid tissue was explored. Six wavelengths (700, 730, 760, 800, 850, 900 nm) and the parameters deoxygenated (HbR), oxygenated (HbO_2_), total hemoglobin (HbT), and saturation of hemoglobin (sO_2_) were analyzed. Ten patients with PA and fourteen with WT were included (12/12 female/male; median age: 51 years). For PA, the mean values for all measured wave lengths as well as for the hemoglobin parameters were different for the tumors compared to the healthy parotid (all p < 0.05). The mean MSOT parameters were all significantly higher (all p < 0.05) in the WT compared to healthy parotid gland except for HbT and sO_2_. Comparing both tumors directly, the mean values of MSOT parameters were not different between PA and WT (all p > 0.05). Differences were seen for the maximal MSOT parameters. The maximal tumor values for 900 nm, HbR, HbT, and sO_2_ were lower in PA than in WT (all p < 0.05). This preliminary MSOT parotid tumor imaging study showed clear differences for PA or WT compared to healthy parotid tissue. Some MSOT characteristics of PA and WT were different but needed to be explored in larger studies.

## Introduction

Salivary gland tumors most commonly occur in parotid glands. Benign tumors accounting for about 75–80%, whereas 20–25% are malignant^1^. The WHO classification list thirteen benign and 20 different malignant phenotypes^2^. Pleomorphic adenoma (PA) and Warthin tumor (WT) are both benign and the most frequent parotid tumors^3^. First sign is in most cases a parotid lump. Malignant parotid tumors can appear very similar to a benign process as they can grow slowly, displacing instead of infiltrating neighboring structures and seem to be mobile. Hence, the clinical examination does not allow a differentiation between the different benign and malignant tumor types in most cases. As most parotid tumors are located in the superficial lobe, the tumors can be visualized with high frequency ultrasound (US)^4^. Newer technologies of contrast-enhanced ultrasound imaging can partially differentiate by showing microvascularization, but not at the molecular level^5–7^. Minimally invasive procedures, in particular fine-needle aspiration (FNA) and large needle biopsy, can also offer a high level of accuracy, but depending on the user and clinic, they are not always highly precise^8^. In addition, the two methods are not used across the board in Germany^9^. Magnetic resonance imaging (MRI; or less likely computed tomography (CT)) are need for larger tumors or in case of signs of malignancy. The major problem is that the specificity of US, MRI, and CT in assessment of the entity of a tumor is moderate^10^. Currently, it is evaluated if techniques like multiparametric MRI or CT-based radiomics can improve the accuracy^11,12^. A good differentiation between the different types of benign and malignant tumor is an important unmet medical need, as it has direct impact on the therapeutic strategy^1^.

Multispectral optoacoustic tomography (MSOT) is an optical imaging technique with high specificity and sensitivity to identify functional and molecular processes in living organisms. MSOT might offer potential solutions to these imaging problems because of its ability to image optical absorption properties of both intrinsic tissue chromophores and exogenous contrast agents without the involvement of ionizing radiation^13^. First data has been published that MSOT allows for differentiation of breast cancer and healthy tissue^14,15^. In a pilot study, MSOT showed different characteristics between benign and malignant thyroid nodules^16^. The aim of the present study was to evaluate the applicability of MSOT to differentiate between the two most frequent benign parotid tumors, PA and WT, and to see which tissue parameters provided by MSOT differentiate these tumors to healthy parotid gland tissue.

## Materials and methods

### Study design and setting

**Figure 1:**
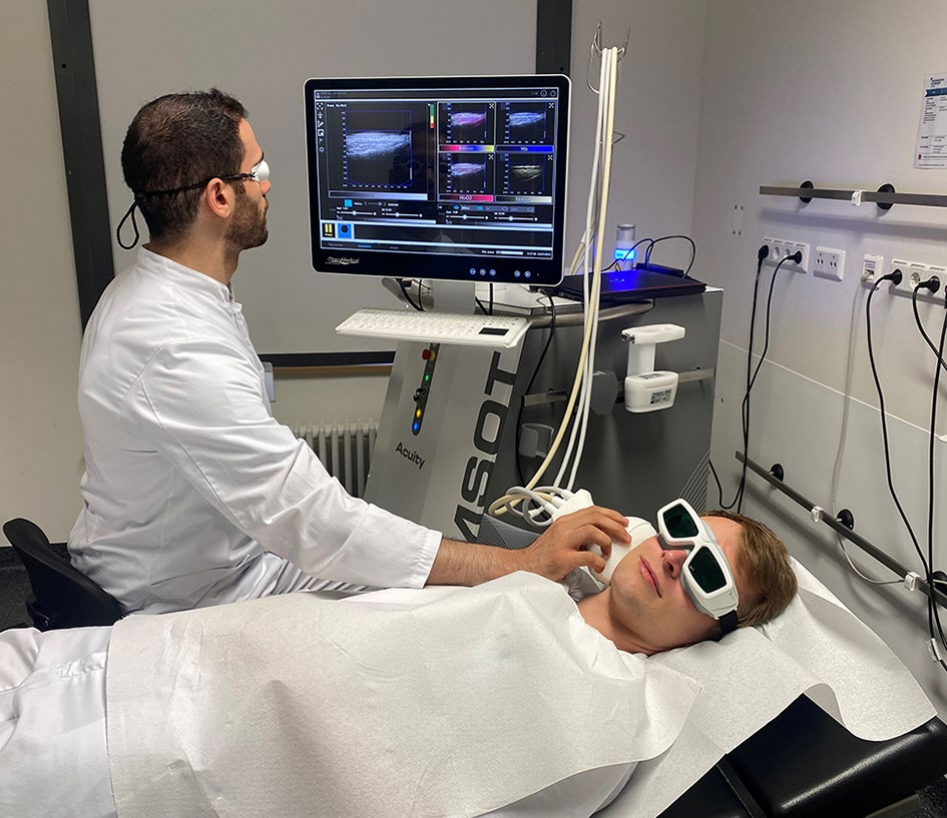
Examination with MSOT of the parotid gland in a clinical setting.

The study was carried out in the BLINDED, between October 2021 and January 2023. The Ethics Committee of the Jena University Hospital approved the study protocol for this prospective observational study (No. 2021-2048-BO). Written informed consent was obtained from all study participants. In addition, informed consent was obtained to publish the facial images in an open-access publication as shown in Fig. 1.

Inclusion criteria were: age ≥ 18 years, unilateral parotid tumor scheduled for parotidectomy, no previous parotid surgery, normal parotid gland on the contralateral side, and histological confirmation of a PA or of a WT. All 24 patients included in our study could be examined. Data on patients’ characteristics were extracted from the patients’ charts. All patients received a standard sonography (MyLabOne, Esaote Biomedica GmbH, Cologne, Germany) of the parotid glands.

### In vivo MSOT imaging

A commercially available handheld MSOT imaging system was used (Acuity Echo; iThera Medical GmbH, Munich, Germany). It consists of an optical oscillator equipped with a Nd: YAG laser and a hemispherical 3D handheld detector. The parotid gland with the tumor and the contralateral parotid gland was imaged. Nanosecond excitation laser pulses were generated at a repetition rate of 25 Hz in a wavelength range of 700, 730, 760, 800, 850 and 900 nm. Multispectral images were acquired using one pulse per wavelength image. A cylindrically focused, 256-element detector array (center frequency, 4 MHz; send/receive bandwidth, 60%; resolution, approximately 190 μm) and 125° coverage provided 2-dimensional cross-sectional images with a field of view of 25 × 25 mm^2^ and a pixel size of 62.5 μm). Laser light was delivered via a fiber bundle (CeramOptec GmbH, Bonn, Germany). Real-time visualization was enabled with viewMSOTc software (v1.2, iThera Medical, Munich, Germany). The recorded images were reconstructed using a standard back-projection algorithm after band-pass filtering and deconvolution with the electrical impulse response of the transducer. More details are described elsewhere^14,17^. To increase the signal-to-noise-ratio, a running average was applied with one sweep consisting of seven sequential frames if no detector movement was determined in the image sequence. The minimal and maximal values were also stored. Reflection ultrasound computed tomography (RUCT)-mode US images were generated by the MSOT device using an US-imaging platform that consolidates transmit-receive boards and a function of triggered acquisition for synchronizing US and OA image streams, as previously described^14,18^.

### MSOT examination procedure

The examination took shorter than 10 min per patient. All examination with the MSOT were performed by one author (M.K.). The examination with MSOT and ultrasound of the parotid gland were performed always preoperatively. The examination with ultrasound were performed by different head and neck surgeons of the department. During the examination with MSOT, the eyes of patient and the examiner were protected with laser safety goggles (Fig. 1). For this purpose, the transducer was held on the examination area for 10 s and a recording was started. The transducer was held still so that no movement artifacts were caused. Several recordings were made for this purpose. Measurements were performed on both parotid glands, i.e. on the side with the parotid tumor and on the contralateral healthy parotid gland. Regions of interest (ROIs) were placed at 0.5–1.5 cm depth based on the US images. Thereby, the ROIs were placed at the superficial tumor margin, which showed an optoacoustic signal. Subsequently, the signals of the contralateral side (which did not show any pathological findings on US) were examined at the same depth. The results of the MSOT examination were expressed in arbitrary units (AU). After parotidectomy, the definitive histopathologic findings could be included in the analyses.

### MSOT image acquisition

All the wavelengths available in the MSOT system were applied. The specific wavelengths allow the measurement of the selected endogenous chromophores. For multispectral imaging, the manufacturer’s presets for 6 wavelengths (700, 730, 760, 800, 850, and 900 nm) were selected indicating results in arbitrary units (AU). The wavelengths mentioned were not selected manually but were defined as default settings by the manufacturer due to the technical limitations of the laser and the possibility to visualize certain chromophores after spectral unmixing. For example, the wavelength 800 nm allows the measurement of the endogenous chromophore of water (H_2_O) and the wavelength 850 nm for the detection of oxygenated hemoglobin (HbO_2_). For hemoglobin (Hb), individual contributions of HbO_2_ and deoxygenated hemoglobin (HbR) were calculated from data acquired and based on their spectral absorption characteristics by spectral unmixing. Total Hb (HbT = HbO_2_ + HbR) and oxygen saturation (sO_2_ = HbO_2_/HbT) were calculated for the selected ROIs co-localized to parotid structures identified on US images using the software iLabs (v.1.3.7, iThera Medical, Munich, Germany). MSOT values for each ROI represent the mean optoacoustic image value of all pixels. Ten MSOT values were calculated for the parotid tumor and the contralateral parotid gland: for six wave lengths, HbR, HbO_2_, HbT, and sO_2_. These MSOT parameters were pseudocolor-coded and visualized individually with the ultrasound image as background in composite images.

### Statistical analysis

Participants’ characteristics and outcome variables were analyzed with IBM SPSS statistics software (Version 28.0.0.0; Chicago, IL, United States) for medical statistics. Data are presented as mean ± standard deviation (SD) if not otherwise indicated. Average values, minimal, and maximal MSOT values of the measurements of the seven sequential frames are presented of the tumor and the contralateral side. In addition, the ratio between tumor and contralateral side was calculated. The chi-square test was used to compare nominal data of the two independent subgroups (PA versus WT). The Mann–Whitney U-test was used to compare scaled data of the two independent subgroups. The Wilcoxon test for paired data was used to compare scaled data of the tumor side with the contralateral side. In general, nominal p values of two-tailed tests are reported. The significance level of p = 0.05 was set.

### Ethical approval

All procedures performed in this study involving human participants were in accordance with the ethical standards of the institutional and/or national research committee and with the 1964 Helsinki declaration and its later amendments or comparable ethical standards. The study was approved by the local ethics committee.

## Results

### Study participants

Patients’ characteristics are summarized in Table 1. Twenty-four patients were included (12 female, 12 male). The mean age was 51.3 ± 16.7 years. Ten patients with a PA and fourteen patients with a WT were included. The diagnosis was confirmed by histopathology of the tumor specimen after parotidectomy. The gender distribution was not different between both benign tumor entities (p = 0.098). The patients with PA were younger than the patients with WT (p = 0.002).

**Table 1:** Patients’ characteristics.

Parameter	All		Pleomorphic adenoma		Warthin tumor		p
	N	%	N	%	N	%	
All	24	100	10	100	14	100	0.098
Gender							
Male	12	50	3	30	9	64.3	
Female	12	50	7	70	5	35.7	

	M ± SD	MD, R	M ± SD	MD, R	M ± SD	MD, R	
Age, years	51.3 ± 16.7	57, 21–74	40.3 ± 16.0	35, 21–72	59.2 ± 12.4	62, 34–74	**0.002**

*M* mean, *SD* standard deviation, *MD* median, *R* range.

Significant p-values (p < 0.05) in bold.

### Comparison of parotid tumors to the contralateral healthy parotid gland

The results of the comparison between the tumor and the contralateral side are presented in the Tables 2 and 3. For PA (Table 2), the mean values for all measured wave lengths as well as for the hemoglobin parameters were different for the tumors compared to the healthy parotid (all p < 0.05). All hemoglobin values were higher in the tumors. There was no difference for the sO_2_ measurements. For the minimal values, the hemoglobin parameters again were higher in the tumor, but also minimal sO_2._ For the spectra, the minimal values for 700 nm, 850 nm, and 900 nm were higher for the tumors. Concerning the maximal values, significant differences were seen only for HbO_2_ and sO_2_ (p < 0.05). In both cases, the tumors showed lower maximal values. The results for WT were different to some content (Table 3). The mean MSOT parameters were all significantly higher in the WT except for HbT and sO_2_. There were no differences between WT and healthy parotid tissue when looking on the minimal MSOT values. The maximal values showed significantly higher spectra for 700–850 nm (not for 900 nm) in the WT compared to healthy parotid tissue.

**Table 2:** Comparison of the multi-spectral optoacoustic tomography measurements (MSOT signal in AU) of pleomorphic adenomas and the normal contralateral parotid gland.

	Tumor side		Contralateral side		p
	Mean	SD	Mean	SD	
ROI, mm^3^	64.15	40.80	64.16	40.81	0.343
Depth, minimal, cm	5.51	1.88	5.90	1.84	0.343
Mean AU values					
700 nm	400.35	126.11	262.73	30.22	**0.008**
730 nm	382.13	128.96	254.03	30.57	**0.010**
760 nm	394.81	124.13	255.42	34.78	**0.005**
800 nm	346.17	111.06	235.87	26.52	**0.010**
850 nm	354.27	112.93	242.12	28.45	**0.010**
900 nm	565.69	241.60	366.76	160.30	**0.001**
HbR	0.18	0.07	0.12	0.02	**0.020**
HbO_2_	0.29	0.12	0.19	0.07	**0.001**
HbT	0.47	0.14	0.31	0.06	**0.003**
sO_2_	0.62	0.11	0.60	0.10	0.350
Minimal AU values					
700 nm	− 61.52	382.84	− 242.59	351.30	**0.049**
730 nm	− 42.16	379.24	− 215.47	356.78	0.055
760 nm	− 13.21	334.69	− 179.96	334.39	0.054
800 nm	− 21.23	337.83	− 172.34	311.84	0.072
850 nm	14.12	277.87	− 146.10	281.76	**0.036**
900 nm	106.18	225.14	− 115.10	251.91	**0.008**
HbR	0.02	0.03	0.00	0.01	**0.033**
HbO_2_	0.07	0.05	0.02	0.02	**0.005**
HbT	0.11	0.09	0.05	0.06	**0.014**
sO_2_	0.37	0.14	0.18	0.14	** < 0.0001**
Maximal values					
700 nm	1014.91	554.26	883.31	492.03	0.187
730 nm	925.72	542.10	840.67	513.57	0.252
760 nm	906.69	495.77	838.73	502.56	0.275
800 nm	784.83	465.09	800.96	482.77	0.439
850 nm	776.88	430.63	797.03	449.77	0.420
900 nm	1144.68	574.99	1097.48	670.48	0.369
HbR	0.49	0.31	0.42	0.24	0.185
HbO_2_	0.62	0.28	1.11	0.44	**0.007**
HbT	1.00	0.51	0.98	0.55	0.442
sO_2_	0.90	0.11	0.98	0.04	**0.023**

*AU* arbitrary unit, *SD* standard deviation, *ROI* region of interest *HbR* deoxygenated hemoglobin, *HBO*_*2*_ oxygenated hemoglobin, *HbT* total hemoglobin, *sO*_*2*_ oxygen saturation.

Significant values are in bold.

**Table 3:** Comparison of the multi-spectral optoacoustic tomography measurements (MSOT signal in AU) of Warthin tumors and the normal contralateral parotid gland.

	Tumor side		Contralateral side		p
	Mean	SD	Mean	SD	
ROI, mm^3^	77.70	43.94	77.70	43.94	0.168
Depth, minimal, cm	5.42	1.96	5.55	1.89	** < 0.0001**
Mean AU values					
700 nm	427.62	84.69	259.90	72.48	** < 0.0001**
730 nm	409.16	75.72	252.51	70.85	** < 0.0001**
760 nm	421.36	67.82	263.91	72.56	** < 0.0001**
800 nm	380.23	58.67	230.80	61.73	** < 0.0001**
850 nm	398.30	66.23	241.32	62.44	**0.001**
900 nm	764.63	356.46	492.99	234.91	** < 0.0001**
HbR	0.17	0.06	0.10	0.05	**0.001**
HbO_2_	0.39	0.17	0.25	0.11	** < 0.0001**
HbT	0.56	0.14	0.35	0.11	0.455
sO_2_	0.68	0.12	0.68	0.13	0.168
Minimal AU values					
700 nm	− 310.26	440.67	− 332.07	361.40	0.379
730 nm	− 276.95	433.67	− 288.31	338.07	0.439
760 nm	− 197.11	388.89	− 226.80	305.10	0.349
800 nm	− 223.84	342.79	− 209.25	245.49	0.425
850 nm	− 167.26	296.43	− 169.25	197.73	0.489
900 nm	− 34.18	449.08	− 182.69	340.05	0.107
HbR	0.00	0.00	0.00	0.01	0.127
HbO_2_	0.03	0.07	0.00	0.00	0.092
HbT	0.04	0.09	0.02	0.03	0.189
sO_2_	0.29	0.22	0.14	0.18	0.008
Maximal AU values					
700 nm	1369.72	739.11	1000.43	532.08	**0.001**
730 nm	1286.58	721.22	966.49	501.59	**0.002**
760 nm	1251.22	632.84	945.89	439.95	**0.002**
800 nm	1125.61	529.38	879.98	411.70	**0.008**
850 nm	1101.35	408.01	906.05	433.61	**0.049**
900 nm	1890.89	958.73	1675.33	1446.20	0.264
HbR	0.64	0.43	0.47	0.29	**0.004**
HbO_2_	1.05	0.46	1.49	0.67	**0.022**
HbT	1.50	0.61	1.27	0.76	0.105
sO_2_	0.99	0.02	0.98	0.06	0.244

*AU* arbitrary unit, *SD* standard deviation, *ROI* region of interest, *HbR* deoxygenated hemoglobin, *HBO*_*2*_ oxygenated hemoglobin, *HbT* total hemoglobin, *sO*_*2*_ oxygen saturation.

Significant values are in bold.

### Comparison of pleomorphic adenoma and Warthin tumor

**Figure 2:**
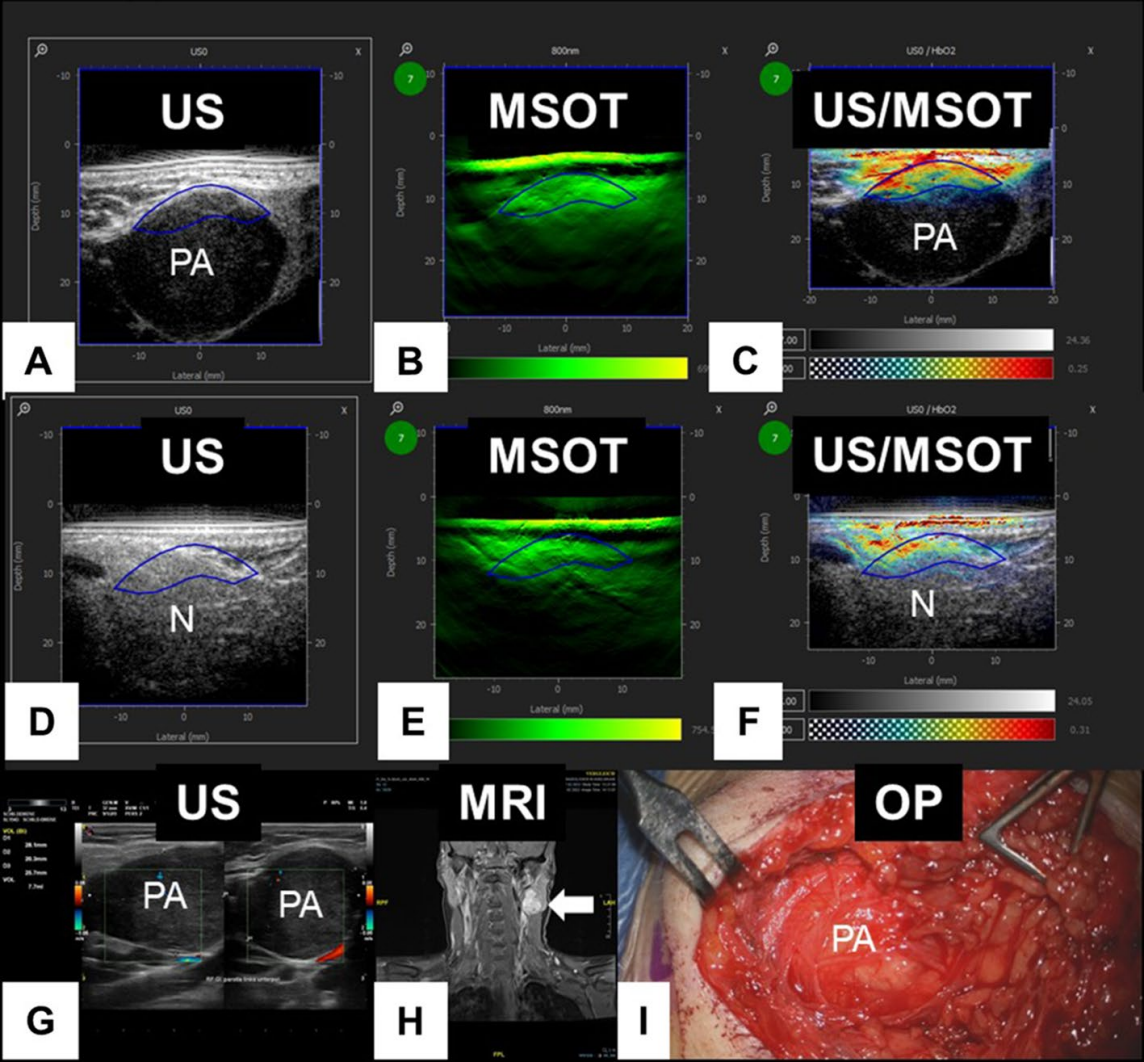
Example of a pleomorphic adenoma (PA) of the left parotid gland. (**A–C**) Parotid gland with tumor; (**D–F**) contralateral healthy parotid gland. (**A**) Ultrasound (US) image of the MSOT system; PA circled in blue. (**B**) MSOT image of 800 nm; Region of interest (ROI) circled in blue. (**C**) Hybrid US/MSOT image; ROI circled in blue. (**D**) US image of the MSOT system; ROI in normal (N) salivary gland tissue circled in blue. (**E**) MSOT image of 800 nm; ROI in normal tissue circled in blue. (**F**) Hybrid US/MSOT image; ROI in normal tissue circled in blue. (**G**) High-resolution image with normal US device. (**H**) Coronal magnetic resonance imaging (MRI) of the neck showing the tumor (arrow) in the parotid gland. (**I**) Intraoperative (OP) situs. The tumor is exposed in the parotid gland.

**Figure 3:**
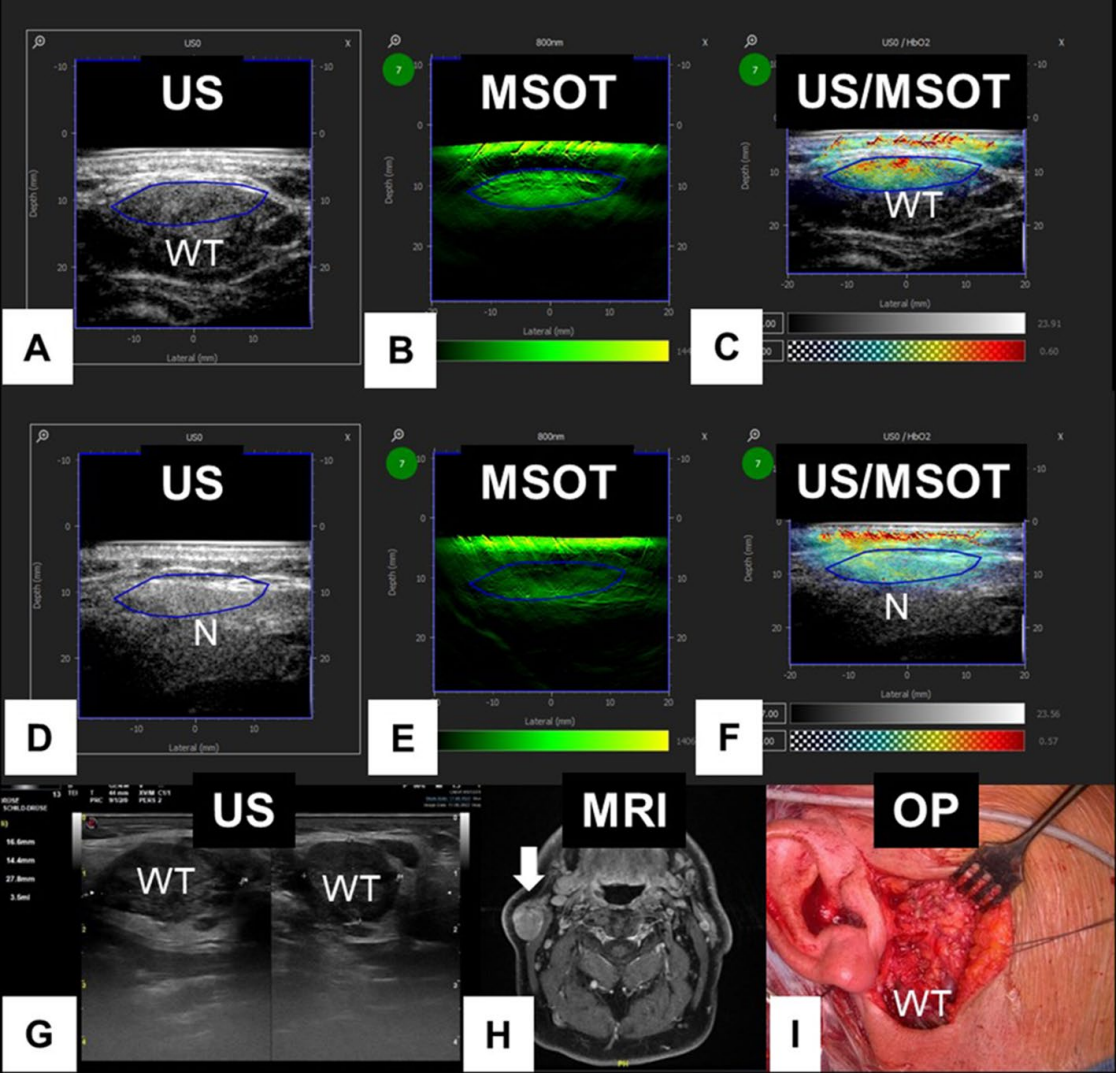
Example of a Warthin tumor (WT) of the right parotid gland. (**A–C**) Parotid gland with tumor; (**D–F**) contralateral healthy parotid gland. (**A**) Ultrasound (US) image of the MSOT system; part of WT circled in blue. (**B**) MSOT image of 800 nm; Region of interest (ROI) circled in blue. (**C**) Hybrid US/MSOT image; ROI circled in blue. (**D**) US image of the MSOT system; ROI in normal (N) salivary gland tissue circled in blue. (**E**) MSOT image of 800 nm; ROI in normal tissue circled in blue. (**F**) Hybrid US/MSOT image; ROI in normal tissue circled in blue. (**G**) High-resolution image with normal US device. (**H**) Magnetic resonance imaging (MRI) showing the tumor (arrow) in the parotid gland. (**I**) Intraoperative (OP) situs. The tumor does is covered by normal parotid tissue and does not only partly reach the surface of the parotid gland.

**Figure 4:**
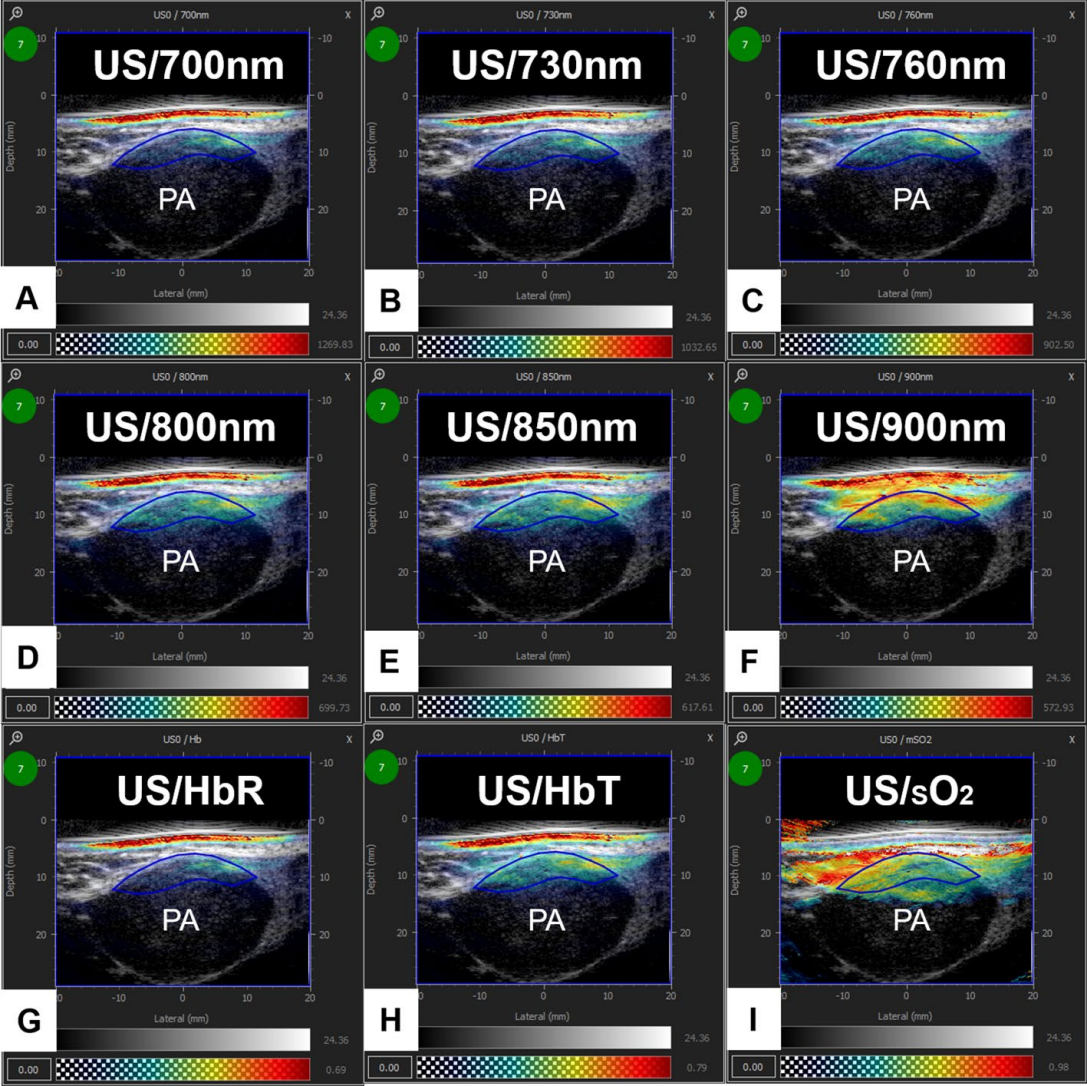
Example of a pleomorphic adenoma (PA) of the left parotid gland with six wavelengths (700, 730, 760, 800, 850, 900 nm) and the parameters deoxygenated hemoglobin (HbR), total hemoglobin (HbT), and saturation of hemoglobin (sO_2_). (**A**) Hybrid US/MSOT image of 700 nm; Region of interest (ROI) circled in blue. (**B**) Hybrid US/MSOT image of 730 nm; ROI circled in blue. (**C**) Hybrid US/MSOT image of 760 nm; ROI circled in blue. (**D**) Hybrid US/MSOT image of 800 nm; ROI circled in blue. (**E**) Hybrid US/MSOT image of 850 nm; ROI circled in blue. (**F**) Hybrid US/MSOT image of 900 nm; ROI circled in blue. (**G**) Hybrid US/MSOT image of HbR; ROI circled in blue. (**H**) Hybrid US/MSOT image of HbT; ROI circled in blue. (**I**) Hybrid US/MSOT image of sO_2_; ROI circled in blue_._

Examples for a PA and WT are presented in Figs. 2 and 3. In addition, examples of a PA of the left parotid gland with six wavelengths (700, 730, 760, 800, 850, 900 nm) and the parameters HbR, HbT and sO2 are shown in Fig. 4. The results of the comparison for the mean, minimal and maximal values are presented in Tables 4, 5 and 6. The mean values of the MSOT parameters (Table 4) were not different on the tumor side, the contralateral side, and for the ratio. Concerning the minimal values (Table 5), there were a few significant differences (all p < 0.05): HbT was higher in PA compared to WT. But minimal values for HbO_2_ and HbT were higher in also on the contralateral side of patients with PA. Concerning the ratio of tumor to contralateral side, only the minimal ratio of the spectrum at 800 nm was higher in PA (p = 0.042). Finally, more differences were seen for the maximal MSOT parameters (Table 6). In the tumor, the values for 900 nm, HbR, HbT, and sO_2_ were lower in PA (all p < 0.05). No differences were seen on the contralateral healthy side. The maximal ratio was only significantly different for one parameter. The maximal sO_2_ ratio was lower in PA compared to WT (p = 0.006).

**Table 4:** Mean values of the multi-spectral optoacoustic tomography measurements (MSOT signal in AU) of the two parotid tumor entities.

	Pleomorphic adenoma		Warthin tumor		p
	Mean	SD	Mean	SD	
Tumor side					
ROI, mm^3^	64.15	40.80	77.70	43.94	0.226
Depth, minimal, cm	5.51	1.88	5.42	1.96	0.457
700 nm	400.35	126.11	427.62	84.69	0.266
730 nm	382.13	128.96	409.16	75.72	0.262
760 nm	394.81	124.13	421.36	67.82	0.253
800 nm	346.17	111.06	380.23	58.67	0.169
850 nm	354.27	112.93	398.30	66.23	0.121
900 nm	565.69	241.60	764.63	356.46	0.070
HbR	0.18	0.07	0.17	0.06	0.363
HbO_2_	0.29	0.12	0.39	0.17	0.054
HbT	0.47	0.14	0.56	0.14	0.064
sO_2_	0.62	0.11	0.68	0.12	0.105
Contralateral side					
ROI, mm^3^	64.16	40.81	77.70	43.94	0.226
Depth, minimal, cm	5.90	1.84	5.55	1.89	0.328
700 nm	262.73	30.22	259.90	72.48	0.454
730 nm	254.03	30.57	252.51	70.85	0.475
760 nm	255.42	34.78	263.91	72.56	0.368
800 nm	235.87	26.52	230.80	61.73	0.405
850 nm	242.12	28.45	241.32	62.44	0.485
900 nm	366.76	160.30	492.99	234.91	0.078
HbR	0.12	0.02	0.10	0.05	0.194
HbO_2_	0.19	0.07	0.25	0.11	0.094
HbT	0.31	0.06	0.35	0.11	0.147
sO_2_	0.60	0.10	0.68	0.13	0.083
Ratio					
ROI, mm^3^	1.00	0.00	1.00	0.00	0.123
Depth, minimal, cm	0.95	0.16	0.98	0.09	0.296
700 nm	1.54	0.52	1.74	0.49	0.170
730 nm	1.51	0.47	1.72	0.46	0.148
760 nm	1.55	0.45	1.70	0.46	0.228
800 nm	1.47	0.45	1.74	0.43	0.081
850 nm	1.47	0.42	1.74	0.46	0.076
900 nm	1.56	0.33	1.67	0.71	0.326
HbR	1.51	0.55	1.81	0.71	0.140
HbO_2_	1.52	0.34	1.73	0.76	0.205
HbT	1.52	0.39	1.70	0.52	0.193
sO_2_	1.02	0.06	1.02	0.14	0.493

*AU* arbitrary unit, *SD* standard deviation, *ROI* region of interest, *HbR* deoxygenated hemoglobin, *HBO*_*2*_ oxygenated hemoglobin, *HbT* total hemoglobin, *sO*_*2*_ oxygen saturation.

**Table 5:** Minimal values of the multi-spectral optoacoustic tomography measurements (MSOT signal in AU) of the two parotid tumor entities.

	Pleomorphic adenoma		Warthin tumor		p
	Mean	SD	Mean	SD	
Tumor side					
ROI, mm^3^	64.15	40.80	77.70	43.94	0.226
Depth, minimal, cm	5.51	1.88	5.42	1.96	0.457
700 nm	− 61.52	382.84	− 310.26	440.67	0.082
730 nm	− 42.16	379.24	− 276.95	433.67	0.091
760 nm	− 13.21	334.69	− 197.11	388.89	0.120
800 nm	− 21.23	337.83	− 223.84	342.79	0.083
850 nm	14.12	277.87	− 167.26	296.43	0.072
900 nm	106.18	225.14	− 34.18	449.08	0.187
HbR	0.02	0.03	0.00	0.00	0.008
HbO_2_	0.11	0.09	0.04	0.09	0.072
HbT	0.37	0.14	0.29	0.22	**0.030**
sO_2_	1014.91	554.26	1369.72	739.11	0.160
Contralateral side					
ROI, mm^3^	64.16	40.81	77.70	43.94	0.226
Depth, minimal, cm	5.90	1.84	5.55	1.89	0.328
700 nm	− 242.59	351.30	− 332.07	361.40	0.276
730 nm	− 215.47	356.78	− 288.31	338.07	0.308
760 nm	− 179.96	334.39	− 226.80	305.10	0.362
800 nm	− 172.34	311.84	− 209.25	245.49	0.374
850 nm	− 146.10	281.76	− 169.25	197.73	0.407
900 nm	− 115.10	251.91	− 182.69	340.05	0.300
HbR	0.00	0.01	0.00	0.01	0.377
HbO_2_	0.02	0.02	0.00	0.00	**0.007**
HbT	0.05	0.06	0.02	0.03	**0.041**
sO_2_	0.18	0.14	0.14	0.18	0.307
Ratio					
ROI, mm^3^	1.00	0.00	1.00	0.00	0.123
Depth, minimal, cm	0.95	0.16	0.98	0.09	0.296
700 nm	− 4.62	12.05	0.19	2.43	0.079
730 nm	4.46	10.65	5.64	21.57	0.438
760 nm	9.55	25.28	− 0.40	1.86	0.076
800 nm	4.75	8.13	− 3.24	12.16	**0.042**
850 nm	3.48	5.94	− 1.11	8.82	0.084
900 nm	1.74	2.09	1.81	3.52	0.476
HbR	3.50		0.00	0.00	
HbO_2_	2.30	1.36	0.00		0.114
HbT	1.84	1.06	0.33	0.58	0.033
sO_2_	2.83	2.59	4.48	6.48	0.254

*AU* arbitrary unit, *SD* standard deviation, *ROI* region of interest, *HbR* deoxygenated hemoglobin, *HBO*_*2*_ oxygenated hemoglobin, *HbT* total hemoglobin, *sO*_*2*_ oxygen saturation.

Significant values are in bold.

**Table 6:** Maximal values of the multi-spectral optoacoustic tomography measurements (MSOT signal in AU) of the two parotid tumor entities.

	Pleomorphic adenoma		Warthin tumor		p
	Mean	SD	Mean	SD	
Tumor side					
ROI, mm^3^	64.15	40.80	77.70	43.94	0.226
Depth, minimal, cm	5.51	1.88	5.42	1.96	0.457
700 nm	1014.91	554.26	1369.72	739.11	0.160
730 nm	925.72	542.10	1286.58	721.22	0.107
760 nm	906.69	495.77	1251.22	632.84	0.098
800 nm	784.83	465.09	1125.61	529.38	0.083
850 nm	776.88	430.63	1101.35	408.01	0.058
900 nm	1144.68	574.99	1890.89	958.73	**0.037**
HbR	0.49	0.31	0.64	0.43	**0.020**
HbO_2_	0.62	0.28	1.05	0.46	0.183
HbT	1.00	0.51	1.50	0.61	**0.007**
sO_2_	0.90	0.11	0.99	0.02	**0.006**
Contralateral side					
ROI, mm^3^	64.16	40.81	77.70	43.94	0.226
Depth, minimal, cm	5.90	1.84	5.55	1.89	0.328
700 nm	883.31	492.03	1000.43	532.08	0.295
730 nm	840.67	513.57	966.49	501.59	0.277
760 nm	838.73	502.56	945.89	439.95	0.292
800 nm	800.96	482.77	879.98	411.70	0.335
850 nm	797.03	449.77	906.05	433.61	0.278
900 nm	1097.48	670.48	1675.33	1446.20	0.127
HbR	0.42	0.24	0.47	0.29	0.337
HbO_2_	0.63	0.34	0.91	0.81	0.160
HbT	0.98	0.55	1.27	0.76	0.165
sO_2_	0.98	0.04	0.98	0.06	0.448
Ratio					
ROI, mm^3^	1.00	0.00	1.00	0.00	0.123
Depth, minimal, cm	0.95	0.16	0.98	0.09	0.296
700 nm	1.32	0.53	1.43	0.40	0.278
730 nm	1.27	0.51	1.39	0.39	0.267
760 nm	1.23	0.47	1.37	0.34	0.209
800 nm	1.09	0.38	1.35	0.39	0.059
850 nm	1.08	0.38	1.34	0.44	0.074
900 nm	1.18	0.43	1.37	0.54	0.185
HbR	1.29	0.53	1.43	0.47	0.248
HbO_2_	1.11	0.44	1.49	0.67	0.064
HbT	1.14	0.41	1.33	0.45	0.153
sO_2_	0.92	0.11	1.01	0.05	**0.006**

*AU* arbitrary unit, *SD* standard deviation, *ROI* region of interest, *HbR* deoxygenated hemoglobin, *HBO*_*2*_ oxygenated hemoglobin, *HbT* total hemoglobin, *sO*_*2*_ oxygen saturation.

Significant values are in bold.

## Discussion

In this pilot study for benign parotid tumors, clinical MSOT was applicable, reproducible, an allowed a distinction between healthy parotid tissue and benign tumor tissue in vivo. As US is the first choice imaging procedure in diagnostics of parotid tumors^14^, the hybrid US/MSOT handheld probe was simple to use in the clinical setting and could be performed within the same timeframe as standard US imaging. Most of the MSOT parameters, the values for all measured wave lengths as well as for the hemoglobin parameters, were different for the benign tumors compared to the healthy parotid tissue. The comparison of the two most frequent benign parotid tumors, PA and WT, showed only some differences, mainly for the maximal MSOT values. Our hypothesis was that there would be more differences between the two types of benign tumors because their histopathological composition is different. We conclude from the MSOT results that at the molecular level, HbO2, HbR and HbT are very similar in these two benign tumors. Future studies should include other wavelengths to see if specific differences between benign tumor types can be found. To our knowledge, this study provides for the first time investigation of parotid tumors with a clinical MSOT.

None of the standard imaging techniques, neither US, MRI nor CT do allow a reliable differentiation between the different types of benign parotid tumors^10^. For differentiation of the tumor entities, cytology or histology is needed^19^. But even minimally invasive procedures especially fine-needle aspiration (FNA) do not have high accuracy in all cases. In addition, it seems that in some countries, for instance in Germany, FNA is be used nationwide because there is not always a cytopathologist available in every hospital for evaluation. At the moment, FNA is used for salivary glands in only about 50% of German hospitals^9^. Therefore, it is of clinical interest to reproduce the present results in larger sample size, as MSOT would then allow a non-invasive differentiation of different tumor entities. As a next step, it would also be of interest to investigate, if MSOT also would allow a differentiation between benign and malignant parotid tumors. Clinically, most malignant tumors can behave for long time like benign tumors^1^. The above mentioned standard imaging technologies also do not allow a reliable distinction between benign and malignant salivary gland tumors. MSOT offers the possibility of assessing physiological and molecular properties of a tumor non-invasively by semi-quantitative assessment of different endogenous chromophores in the tissue like hemoglobin, melanin, lipids etc^13^. Common imaging technologies do not allow to analyze microvascular changes. There are new technologies of contrast-enhanced ultrasound imaging that can show microvascularization, but not at the molecular level^5,7^. It is a strength of MSOT to display microvascularity and tissue oxygenation by hemoglobin absorption of multiple wavelengths of light to generate high optoacoustic contrast^20^. In the present study, MSOT could distinguish between oxygenation states of hemoglobin, allowing visualization of differential blood saturation by oxygen within tumor and normal parotid tissue. The aim of this pilot study was to evaluate the applicability of MSOT in parotid gland tumors. The comparison of the two most common benign parotid gland tumors, PA and WT showed only some differences, mainly in the maximum MSOT values. At present, the MSOT examination does not offer any direct clinical added value compared to an ultrasound examination for the differentiation of the two most frequent benign parotid tumors. Like in the present study, MSOT based on oxyhemoglobin could also distinguish between breast cancers and other breast abnormalities^21^. In the same way, identical MSOT parameters allowed a differentiation of different benign and malignant thyroid disorders^16^. There are several biophotonic procedures are explored for diagnostic use in head and neck surgery^22^. In literature, there are so far only isolated few investigations on salivary glands using other biophotonic methods such as optical coherence tomography (OCT) or Raman spectroscopy^23,24^. In general, other biophotonic procedures have some limitations compared to MSOT. Some need a contrast agent and have a low penetration depth. MSOT has the advantage of being able to detect endogenous chromophores without the use of markers and can also be combined with dyes or nanoparticles. In addition MSOT also has its strength in penetration depth relative to molecular resolution. No other biophotonic method achieves a penetration depth of 3–4 cm.

The present study has several limitations. The small sample size and restriction to PA and WT do not allow a generalization of the results to all parotid tumors. MSOT has a much better penetration depth (of several centimeters) compared to other in vivo biophotonic technologies. Nevertheless, it is important to notice that larger parotid tumors (cf. Figs. 1, 2) could not be visualized completely with MSOT. Moreover, it is known that MSOT shows with increasing tissue depth an reduction of mean MSOT values and greater spread due to light scattering and absorption^16^. Therefore, we had to limit the ROIs for the analysis of the parotid tumors to the superficial tumor parts. For the clinical use of MSOT imaging in parotid tumor or head and neck cancer, the limitations such as depth penetration and the associated difficulties in examining deeper anatomical regions must be overcome. A further limitation of this pilot study was the limited number of six wavelengths used for data acquisition. Newer but still pre-clinical developments allow the application of significantly more wavelengths within an acceptable time window, resulting in more reliable spectral unmixing^16^. In general, MSOT is still prone to several artefacts originating e.g. from the limited view of the probes and from perturbations from overlaying tissue as for instance large blood vessels producing high MSOT signal^16^.

Next step, we will extend the study to malignant tumors and will enlarge the sample size. We will investigate in a larger study why there were differences especially in the maximum MSOT values. Furthermore, we will hopefully profit from current advances in image reconstruction and unmixing algorithms, for instance realized by segmantic segmentation of the MSOT images using deep learning methods^25^. Additionally, we would like to explore the additive value of the application of indocyanine green (ICG) as a special tracer. ICG is one of the most commonly used fluorophores in near-infrared fluorescence-guided techniques^26^. MSOT can visualize the kinetics of ICG uptake and clearance in the vasculature of tumors in real-time^27^. Other tracers to be applied together with MOST are not yet available for clinical use. We hypothesize that the intravenous application of ICG before MSOT imaging will discover a greater additive value for the discrimination between different types of benign parotid gland tumors and will allow us an even better discrimination between different benign and malignant parotid tumor types.

## Conclusion

The present pilot study demonstrated how MSOT offers several compelling features for parotid tumor diagnostics that are not available using other imaging methods without invasive procedures. These features need to be explored in larger trials and multicenter settings.

## Data Availability

The datasets used and/or analysed during the current study available from the corresponding author on reasonable request.
